# Incorporation of Toll-Like Receptor Ligands and Inflammasome Stimuli in GM3 Liposomes to Induce Dendritic Cell Maturation and T Cell Responses

**DOI:** 10.3389/fimmu.2022.842241

**Published:** 2022-02-18

**Authors:** Maarten K. Nijen Twilhaar, Lucas Czentner, Rianne G. Bouma, Katarzyna Olesek, Joanna Grabowska, Aru Zeling Wang, Alsya J. Affandi, Saskia C. Belt, Hakan Kalay, Cornelus F. van Nostrum, Yvette van Kooyk, Gert Storm, Joke M. M. den Haan

**Affiliations:** ^1^ Department of Molecular Cell Biology and Immunology, Cancer Center Amsterdam, Amsterdam Infection and Immunity Institute, Amsterdam University Medical Center, Vrije Universiteit Amsterdam, Amsterdam, Netherlands; ^2^ Department of Pharmaceutics, Faculty of Science, Utrecht University, Utrecht, Netherlands; ^3^ Department of Biomaterials Science and Technology, Faculty of Science and Technology, University of Twente, Enschede, Netherlands; ^4^ Department of Surgery, Yong Loo Lin School of Medicine, National University of Singapore, Singapore, Singapore

**Keywords:** liposomes, toll-like receptor ligand, inflammasome stimuli, dendritic cells, T cells, vaccination, cancer

## Abstract

Cancer vaccination aims to activate immunity towards cancer cells and can be achieved by delivery of cancer antigens together with immune stimulatory adjuvants to antigen presenting cells (APC). APC maturation and antigen processing is a subsequent prerequisite for T cell priming and anti-tumor immunity. In order to specifically target APC, nanoparticles, such as liposomes, can be used for the delivery of antigen and adjuvant. We have previously shown that liposomal inclusion of the ganglioside GM3, an endogenous ligand for CD169, led to robust uptake by CD169-expressing APC and resulted in strong immune responses when supplemented with a soluble adjuvant. To minimize the adverse effects related to a soluble adjuvant, immune stimulatory molecules can be incorporated in liposomes to achieve targeted delivery of both antigen and adjuvant. In this study, we incorporated TLR4 (MPLA) or TLR7/8 (3M-052) ligands in combination with inflammasome stimuli, 1-palmitoyl-2-glutaryl-sn-glycero-3-phosphocholine (PGPC) or muramyl dipeptide (MDP), into GM3 liposomes. Incorporation of TLR and inflammasome ligands did not interfere with the uptake of GM3 liposomes by CD169-expressing cells. GM3 liposomes containing a TLR ligand efficiently matured human and mouse dendritic cells *in vitro* and *in vivo*, while inclusion of PGPC or MDP had minor effects on maturation. Immunization with MPLA-containing GM3 liposomes containing an immunogenic synthetic long peptide stimulated CD4^+^ and CD8^+^ T cell responses, but additional incorporation of either PGPC or MDP did not translate into stronger immune responses. In conclusion, our study indicates that TLRL-containing GM3 liposomes are effective vectors to induce DC maturation and T cell priming and thus provide guidance for further selection of liposomal components to optimally stimulate anti-cancer immune responses.

## Introduction

Tumor cells are derived from healthy cells and contain multiple somatic mutations due to, for example, errors in DNA replication, failure in DNA repair and exposure to carcinogens (1). As mutations accumulate and the malignant transformation of tumor cells gradually develops, there is a progressive loss in classification of these cells as ‘self’. Indeed, malignant transformation coincides with the expression of tumor antigens, that can either be aberrantly expressed proteins, overexpressed proteins or newly developed tumor-specific antigens (2). Subsequently, the immune system can respond to these ‘non-self’ malignantly transformed cells, leading to tumor cell killing, especially by CD8^+^ T cells (3). The efficacy of immunotherapies such as immune checkpoint inhibition (ICI) illustrates the power of the immune system in (re-)enforcing the anti-tumor response (4). However, clinical responses are not observed in all patients and can be linked to pre-existing immune responses (5). Since cancer vaccination aims to induce antigen-specific T cell responses in patients, it is expected that combining ICI with vaccination will synergistically enforce the immune system and thereby will improve clinical outcome (6–8).

A prerequisite for a successful cancer vaccine lays in the activation of dendritic cells (DC) to prime T cells. DC can be activated by pathogen-associated molecular patterns, such as Toll-like receptor (TLR) agonists, which results in cellular maturation and coincides with upregulation of costimulatory molecules and cytokine secretion (9). Indeed, DC maturation is pivotal for effective cancer vaccination, as T cell priming in the absence of co-stimulation results in tolerance induction (9). Coinciding with DC maturation, vaccine antigen can be processed and subsequently cross-presented in MHC class I or in MHC class II to activate CD8^+^ or CD4^+^ T cells, respectively (10). Interestingly, stimulation of DC with TLR agonists increases the capacity of DC to cross-present co-delivered antigen to CD8^+^ T cells (11, 12). Therefore, co-delivery of antigen and adjuvant by means of a carrier is an interesting approach to maximize immune responses to the vaccine antigen. TLR can reside embedded within the plasma membrane or in endosomal compartments, which correlates with the nature of their ligands (13). In addition to TLR, also various innate cytosolic receptors such as retinoic acid-inducible gene I (RIG-I), melanoma differentiation-associated protein 5 (MDA5) and nucleotide-binding oligomerization domain-containing protein 2 (NOD2) can induce cellular maturation upon recognition of (viral) RNA (RIG-I and MDA5) or bacterial peptidoglycans (NOD2) (14). Various types of nanoparticles can be used as vaccine vector. Among others, liposomes received attention because of their versatile nature which allows them to transfer both hydrophilic as well as hydrophobic cargo (15). Furthermore, liposomes can be surface-decorated with targeting moieties, to facilitate cellular targeting (16, 17). Thus, by use of liposomes and combining both antigen and adjuvant in a single particle that is targeted to a cell of interest *in vivo*, the potency of cancer vaccination is expected to be augmented (18–20).

DCs are the only cells that can stimulate naïve T cell responses, but also consist of different subsets that differ in their capacity to (cross-)present antigen (21–23). DC can be broadly divided into conventional DC1 (cDC1), cDC2 and plasmacytoid DC (pDC) (24). Of these subsets, the transcription factor *Batf3*-dependent cDC1 is especially pivotal in the anti-cancer responses (25). Indeed, high numbers of cDC1 in the tumor microenvironment is associated with favorable prognosis (26). Within the tumor microenvironment, cDC1 secrete various chemokines that are important for T cell attraction and survival (26, 27). Importantly, cDC1 have a superior capacity to recognize dying cells and to cross-present antigen and thereby activate CD8^+^ T cells, as compared to other DC subtypes (21–23, 28, 29). In addition, it is becoming increasingly clear that cDC1 can also play a pivotal role in the initiation of CD4^+^ T cell responses (30, 31). Because of these traits, cDC1 are considered to be an attractive target for various cancer vaccination strategies (23, 32–34).

A recently described alternative cellular target for cancer vaccines are CD169-expressing macrophages (18). These cells are strategically located in the spleen to capture particulate antigen and marked by their high expression of CD169, also known as Siglec-1 or sialoadhesin (35–38). This adhesion molecule is involved in sequestering pathogens such as human immunodeficiency virus (HIV) as well as SARS-CoV-2 (39–42). Importantly, upon capture of particulate antigen, CD169-expressing macrophages were found to efficiently transfer vaccine antigen to cDC1 to elicit an immune response, as observed for antibody-mediated targeting of antigen to CD169-expressing macrophages (43, 44). The natural ligand of CD169 are α2,3-linked sialic acids that are present in gangliosides incorporated in viruses and these gangliosides also facilitate targeting of artificial viral nanoparticles to these cells (45–47). We have recently shown that inclusion of the ganglioside GM3 in antigen-containing liposomes resulted in robust targeting to CD169-expressing macrophages and, when co-injected with a strong soluble adjuvant (activating anti-CD40 antibody and poly IC), induced CD8^+^ T cell responses that were dependent on cDC1 (48, 49). In addition to the endogenous ligands for the receptor, CD169-expressing macrophages could also be targeted with liposomes that harbored a synthetic ligand for CD169 (50, 51). Hence, targeting vaccine components to CD169-expressing macrophages is an attractive strategy to induce cDC1 activation and subsequent adaptive immune responses.

Various strategies are employed in the design of nanovaccines to activate the immune system and TLR agonists are ubiquitously used to induce cellular maturation (19, 20). Recently, it was also noted that low level inflammation, caused by cellular exposure to oxidized lipids in combination with a TLR agonist, induces robust T cell responses through the induction of a hyperactive state of DC (52). However, the precise role of oxidized lipids in cellular maturation and cytokine secretion is not necessarily beneficial, as a link between exposure to oxidized lipids and diminished immune responses was also observed (53). Thus, the effect of oxidized lipid may be dependent on tissue context and time, while the nature of the recipient cell type also appears to play a role (54). In addition to oxidized lipids, beneficial synergistic effects on cellular maturation were observed when muramyl dipeptide (MDP), a ligand for NOD2, was combined with a ligand of TLR4 or 7/8 (55, 56). Mechanistically, both oxidized lipids and MDP can directly or indirectly, induce activation of the inflammasome, without causing cellular death by pyroptosis (52, 55, 56). Thus, combining TLR ligands with inflammasome stimuli may be an attractive approach to harness anti-cancer immunity, without the need to supplement antigen-containing liposomes with a strong soluble adjuvant. In this work, we prepared liposomes that contain the targeting moiety GM3 and a TLR ligand plus an inflammasome ligand or a combination of one TLR ligand and one inflammasome ligand. We assessed to what extent dual liposomal delivery of TLR ligands and/or inflammasome ligands to CD169-expressing cells aid cellular maturation of human and mouse DCs *in vitro* and *in vivo*. Moreover, we assessed how these adjuvants affect the magnitude of the T cell response as compared to the strong soluble adjuvant (activating anti-CD40 antibody and poly IC) we used earlier (48, 49). These data indicate that GM3/TLR ligand-containing liposomes are suitable vectors for cancer vaccines, but that combined stimulation of TLR and inflammasome components *via* CD169-targeted GM3-containing liposomal delivery yields minor additive effects on the magnitude of the T cell response.

## Materials and Methods

### Liposome Preparation and Characterization

Liposomes were prepared from a mixture of phosphatidylcholine, phosphatidylglycerol (Lipoid GmbH, Ludwigshafen, Germany) and cholesterol (Sigma Aldrich, Darmstadt, Germany) in a 3.8:1:2.5 molar ratio, dissolved in methanol/chloroform (2:1). Subsequently, 15 µmol of the lipid mixture was transferred to a round bottom flask. Where indicated, supplemented with GM3 (3 mol%) (Merck, Darmstadt, Germany), telratolimod/3M-052 (2 mol%) (Bio-connect, Huissen, the Netherlands), monophosphoryl lipid A (MPLA) (2 mol%) (Merck, Darmstadt, Germany), 1-palmitoyl-2-glutaryl-sn-glycero-3-phosphocholine (PGPC) (2 mol%) (Merck, Darmstadt, Germany) and/or L18-MDP (2 mol%) (*In vivo* Gen, Toulouse, France). Additionally, 0.1 mol% of the lipophilic fluorescent tracer DiD (1′-dioctadecyl-3,3,3′,3′-tetramethyl indodicarbocyanine, Life Technologies, Frederick, MD, USA) was incorporated. Next, the organic phase was evaporated under reduced pressure using a rotavapor and lipid films were hydrated in 10 mM HEPES buffer pH 7.4. Where indicated, this buffer contained 1 mg/ml of immunogenic OVA_247–279_-peptide (produced in house). Liposomes were sized through stacked polycarbonate filters, 400/200 nm, using high pressure nitrogen and sequentially concentrated and washed from non-encapsulated peptide by ultracentrifugation (200,000*g*, Beckman Coulter). The liposome pellet was resuspended in HEPES buffer as described above. The phosphate content, liposomal mean size, polydispersity index (PDI), and zeta-potential were determined as previously described (49).

### CD169 Fc ELISA

Liposomes were diluted in ethanol to a final concentration of 25 µM phospholipids and coated on Immuno MaxiSorp plates (NUNC, Roskilde, Denmark). 1% BSA (BSA; Fraction V, Fatty acid free, Calbiochem, San Diego, CA, USA) diluted in PBS was used to block coated plates. Next, samples were incubated with CD169 Fc or its mutant form (CD169 Fc R97A) (2 μg/mL) for 1 hour at room temperature (kindly provided by Prof. Dr. P.R. Crocker, University of Dundee) (57). Then, peroxidase-conjugated goat anti-human IgG (Jackson ImmunoResearch, Ely, UK) was added for an additional 30 minutes and plates were washed, TMB (Sigma Aldrich, Darmstadt, Germany) was added as a substrate and the optical density (OD) measured in a microplate absorbance spectrophotometer (Biorad, Hercules, CA, USA) at 450 nm.

### Liposome Uptake, Cytokine Secretion and Maturation by Monocyte-Derived DC (moDC)

Human peripheral blood mononuclear cells (PBMC) were isolated from buffy coats (Sanquin, Amsterdam, the Netherlands), as described before (17). Briefly, blood was mixed with 1% citrate-containing PBS, carefully layered on top of Lymphoprep (Alere Technologies AS, Oslo, Norway) and centrifuged for 30 minutes at 800*g.* Next, monocytes and lymphocytes were collected and washed in 1% citrate in PBS. In order to separate monocytes from contaminating lymphocytes, PBMC were added to a Percoll (GE Healthcare, Chicago, USA) layer and centrifuged for 10 minutes at 300*g.* Monocytes were collected and washed three times in 1% citrate in PBS. Subsequently, monocytes were resuspended in RPMI supplemented with 10% FCS (Biowest, Manassas, VA, USA), 50 U/mL penicillin (Lonza, Basel, Switzerland), 50 μg/mL streptomycin (Lonza, Basel, Switzerland) and 500 μg/mL IL-4 (ImmunoTools, Friesoythe, Germany) and 800 μg/mL Granulocyte Macrophage Colony stimulating Factor (GM-CSF) (ImmunoTools, Friesoythe, Germany) and cultured for 6 days to generate moDC. Where indicated, moDC were pretreated with type I interferon (IFN) (100 IU/mL, or dose as indicated in the figure) to upregulate the expression of CD169 on day 4. For liposome uptake and maturation moDC were plated and 100 μM of phospholipid (diluted in full medium) were added to the moDC. After 45 minutes the cells were washed two to three times and resuspended in fresh medium. After 15-18 hours the supernatant was collected for an IL-1β ELISA, according to the manufacturers’ protocol (Biolegend, San Diego, CA, USA), the cells were stained and analyzed as described under ‘flow cytometry’. Where indicated, a CD169-blocking antibody (10 μg/ml clone 7.239) or an isotype control anti-Langerin [10 μg/ml clone 10E2 (produced in house)], was added to the moDC 15 minutes prior to liposome incubation. For intracellular cytokine detection, moDC were plated and incubated with 100 μM of phospholipid for 2 hours, subsequently cells were extensively washed and incubated in fresh medium for an additional 3 hours in the presence of Golgiplug, (BD, Bioscience, San Jose, CA, USA). Cells were then stained and analyzed as described under ‘flow cytometry’. Individual donors were represented by individual data points in the figures.

### Animal Experiments

C57BL/6 WT mice were bought from Charles River or bred in house. Male and female mice between 8 and 24 weeks of age were used for maturation studies. Female mice between 8 and 12 weeks were used for T cell priming experiments. These experiments were approved by the National Committee for Animal Experiments (CCD AVD1140020171024, 10 April 2017) and the local animal welfare body, Vrije Universiteit, Amsterdam UMC.

### *In Vivo* Maturation and T Cell Priming

Mice were intravenously (i.v.) injected with liposomes (dose as indicated in figure legend, when not specified, 200 nmol phospholipid). Where indicated, liposomes were replaced or supplemented with 25 µg of poly IC (low molecular weight (LMW), *Invivogen*, Toulouse, France) and 25 µg of activating anti-CD40 antibody (aCD40) (clone 1C10, produced in house), as positive control for DC maturation or T cell priming. Spleens were collected 16 hours or 7 days after the injection, for DC maturation or T cell priming, respectively. Splenocytes were either restimulated with short peptides for 5 or 25 hours [during the last 5 hours in the presence of Golgiplug, (BD, Bioscience, San Jose, CA, USA)], for CD8^+^ and CD4^+^ T cells, respectively and subsequently used for FACS analysis. Alternatively, splenocytes were directly used for flow cytometry analysis. Individual mice were indicated as single data points in the figures.

### Splenic Digestion

Murine spleens were enzymatically digested when macrophages or DC were analyzed. Briefly, spleens were mechanically dissociated and digested in a mixture of 3 mg/mL lidocaine, 2 WU/mL Liberase TL (Roche, Mannheim, Germany) and 50 mg/mL DNase (Roche, Mannheim, Germany) for 12 minutes at 37°C, while the mixture was continuously stirred. Next, ice-cold medium (RPMI-1640 (Gibco, Life Technologies, Paisley, UK) supplemented with 10% FCS, 10 mM EDTA, 20 mM HEPES and 50 μM 2-mercaptoethanol (Gibco, Life Technologies, Paisley, UK) was added, after which the digestion continued for 10 minutes at 4°C. Red blood cells were lysed using an ammonium-chloride-potassium lysis buffer and remaining splenocytes were filtered through a 70-100 μm filter.

For T cell assays, spleens were mechanically dissociated by mashing the spleen through an 70-100 μm filter, after which red blood cells were lysed using an ammonium-chloride-potassium lysis buffer.

### *In Vitro* Liposome Incubation

A single cell suspension of splenocytes was incubated with adjuvant-containing liposomes, 100 μM phospholipid (diluted in full medium), or with soluble adjuvants (dose equal to 2 mol% present in liposomes which is 5 µg/mL for TLR4L, 2 µg/mL for TLR7/8L, 2 µg/mL for PGPC or 3 µg/mL for MDP). After 45 minutes at 37°C splenocytes were extensively washed and surface stained (as described below), to asses liposome uptake. Alternatively, cells were extensively washed and incubated overnight at 37°C to mature, after which staining was performed.

### Flow Cytometry

MoDC were washed and incubated with human Fc block (BD, Bioscience, San Jose, CA, USA) in the presence of the Fixable Viability Dye eFluor 780 (eBioscience, San Diego, CA, USA), diluted in 0.5% BSA in PBS. Next, moDC were stained with an anti-CD86 FITC conjugated antibody (clone BU63) (ImmunoTools, Friesoythe, Germany) for 30 minutes at 4°C. Subsequently, cells were fixed in 2% PFA (Electron Microscopy Sciences, Hatfield, PA, USA), washed and resuspended in 0.5% BSA in PBS until FACS analysis. When intracellular targets (IL-1β, clone REA577, Miltenyi Biotec, Leiden, the Netherlands) were analyzed, cells were washed in 0.5% saponin buffer after fixation and stained for 30 minutes at 4°C.

A single cell suspension of splenocytes was incubated with 10 μg/mL of Fc block (anti CD16/32, clone 2.4G2, produced in house) for 15 minutes with the Fixable Viability Dye eFluor 780, diluted in 0.5% BSA in PBS. If applicable, a tetramer staining (H-2K^b^/OVA_257-264_ (kindly provided by K.L. Franken, Dept of Immunology, LUMC) and I-A^b^/OVA_262-276_ (kindly provided by the Tetramer core facility, National Institute of Health (NIH), Atlanta, GA, USA) was performed for 45 minutes at 37°C, followed by a surface staining. Cells were surface stained with the antibodies listed below for 30 minutes at 4°C (**Table 1**). After surface staining, cells were fixed in 2% PFA. For staining of intracellular targets, cells were subsequently permeabilized with 0.5% saponin buffer and stained for 30 minutes at 4°C (**Table 1**).

**Table 1:** Anti-mouse antibodies.

Antigen/reagent	Fluorochrome	Clone	Company	Panel
XCR1	BV421	ZET	Biolegend	Maturation
I-A/I-E	BV510	M5/114.15.2	eBioscience	
CD40	Biotin/SA-605	1C10	In house made	
CD11c	BV650	HL3	BD Biosciences	
BST2	BV711	129C1	Biolegend	
CD11b	BV786	M1/70	Biolegend	
Ly6G	Percp-Cy5.5	1A8	Biolegend	
CD169	Alexa Fluor 488	SER-4	In house made	
CD80	PE	16-10A1	Immunotools	
F4/80	PE-CF594	T45–2342	BD Biosciences	
Lineage (CD3e, CD19 and NK1.1)	PE-Cy5	145-2C11, 6D5, PK136, respectively	Biolegend	
CD86	PE-Cy7	53-6.7	BD Biosciences	
Sirpa	AF700	P84	Biolegend	
CD8a	PE-Cy7	53–6.7	BD Biosciences	T cell tetramer staining
CD4	PerCP-Cy5.5	RM4-5	eBioscience	
CD44	FITC	KM81	Immunotools	
H-2Kb/OVA_257-264_	PE tetramer	N/A	Leids universitair medisch centrum (LUMC), Leiden, the Netherlands	
I-Ab/OVA_262-276_	APC tetramer	N/A	Tetramer core facility NIH, Atlanta, GA, USA	
CD127	BV510	A7R34	Biolegend	
PD-1	BV785	29F.1A12	Biolegend	
CD62L	AF700	MEL-14	Biolegend	
KLRG1	ef450	2F1	eBioscience	
CD11a	FITC	M17/4	eBioscience	Re-stim intracellular IFNγ staining
CD8a	PE-Cy7	53–6.7	BD Biosciences	
CD3e	PE-Cy5	145-2C11	Biolegend	
CD4	PE	GK1.5	eBioscience	
IFNγ	APC	XMG1.2	eBioscience	

### Statistical Analysis

The statistical analysis was performed using GraphPad Prism v8 (GraphPad, San Diego, CA, USA). A one-tailed t-test was used to determine statistical differences between two groups. For three groups or more, a one-way ANOVA with a Tukey *post-hoc* test was used to determine statistical differences. Alternatively, when differences were compared to a control formulation, a Dunnett *post-hoc* test was used. When near significance was reached (*i.e.* a p-value of 0.1, this was indicated). The p-value of 0.05 or lower was considered significant.

## Results

### Inclusion of TLR and Inflammasome Agonists Does Not Alter Physicochemical and CD169 Binding Properties of GM3 Liposomes

**Figure 1:**
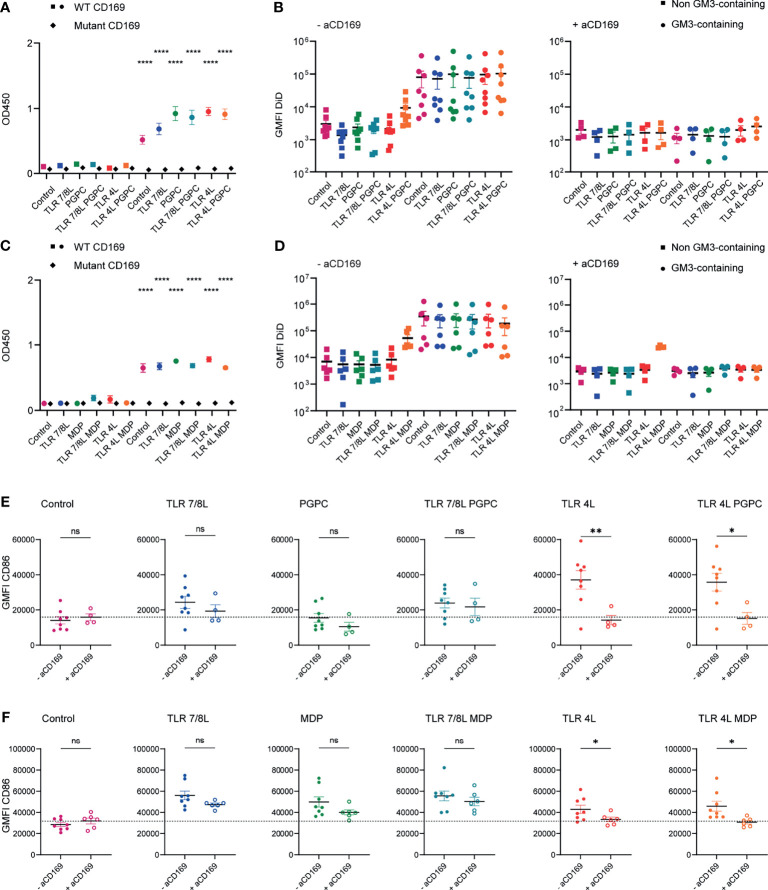
Adjuvant inclusion in GM3 liposomes does not alter binding properties of liposomes and TLRL-inclusion induces moDC maturation. **(A)** Liposomes were coated on an ELISA plate and the organic solvent was evaporated overnight. Subsequently, binding to WT or mutant CD169 was determined. Indicated is the average ± SEM of three independent experiments, performed in triplicate. Significance was compared to the non-GM3-containing counterpart. **(B)** Liposomes were incubated with moDC at 37°C, that were untreated or pretreated with a blocking antibody 15 minutes prior to liposome incubation. Indicated is the average DiD-signal ± SEM (*n* = 4-8). **(C)** As in A, indicated is the average ± SEM of a technical triplicate. Significance was compared to the non-GM3-containing counterpart. **(D)** Liposomes were incubated with moDC (that were pretreated with type I IFN) at 37°C, that were untreated or pretreated with a blocking antibody 15 minutes prior to liposome incubation. Indicated is the average DiD-signal ± SEM (*n* = 4-6). **(E)** MoDC were non-treated or incubated with a blocking antibody for 15 minutes and subsequently incubated with liposomes for 45 minutes at 37°C. Next, unbound liposomes were washed away and cells were incubated for 15-18h at 37°C, after which the cells were stained and the expression of CD86 was determined by flow cytometry analysis. Indicated is the average geometric mean fluorescent intensity (GMFI) of CD86 ± SEM (*n* = 4-6). **(F)** as in E, but cells were pretreated with type I IFN (100 IU/mL). Indicated is the average GMFI of CD86 ± SEM (*n* = 6-8). *p < 0.05, **p < 0.01, ****p < 0.0001. ns, not significant.

In order to induce cellular maturation *via* liposomal adjuvant delivery, we used two different Toll-like receptor (TLR) agonists and two previously described inflammasome agonists (52, 55, 56). We prepared two series of liposomes in which we combined a TLR agonist (telratolimod/3M-052 for TLR7/8 or MPLA for TLR4) with the inflammasome stimulus oxidized lipid 1-palmitoyl-2-glutaryl-sn-glycero-3-phosphocholine (PGPC), or nucleotide-binding oligomerization domain-containing protein 2 (NOD2) ligand L18-MDP (further referred to as MDP). Liposomes contained either one TLR ligand or a combination of one TLR ligand combined with one inflammasome stimulus. As controls, we prepared liposomes that did not contain any adjuvant (referred to as control), or contained only the oxidized lipid PGPC or only MDP (**Supplementary Figures 1A, B**). In addition, liposomes were without targeting ligand or contained the ganglioside GM3 (annotated with squares or circles, respectively) to facilitate specific uptake by CD169-expressing cells (18, 48, 49, 58, 59). The liposomes exhibited a similar mean size, in the range of 160-190 nm and a PDI below 0.2, indicating a relatively homogenous size distribution and the zeta-potential was negative, around -50 mV (**Supplementary Figures 1A, B**). All GM3 liposomes, but not the liposomes lacking GM3, bound to recombinant mouse CD169 in an ELISA-based assay (**Figures 1A, C**). We additionally investigated the binding of liposomes to the mutant form of mouse CD169 that contains an amino acid substitution (R97A), rendering it incapable of specific ligand binding (57). Since none of the formulations bound to the mutant form of CD169, we concluded that GM3 liposomes specifically bind CD169 (**Figures 1A, C**). Thus, adjuvant inclusion in liposomes did not alter the physicochemical properties of the liposomes and all GM3 liposomes bound specifically to CD169.

### TLR-Ligand-Containing GM3 Liposomes Bind to Human Monocyte-Derived Dendritic Cells and Induce Maturation

GM3 liposomes bind to both human and mouse CD169-expressing cells (18, 48, 49, 58, 59). We first determined the capacity of the liposomes to be taken up and to activate human CD169-expressing APCs. We generated monocyte-derived dendritic cells (moDC) and where indicated, moDC were pretreated with 100 IU/mL type I IFN, to upregulate CD169 (58), which did not affect the expression of the maturation marker CD86 (**Supplementary Figure 1C**). We incubated liposomes with moDC for 45 minutes at 37°C, and we included a blocking antibody to CD169 to determine the specificity for the CD169 receptor as moDCs also express other Siglec molecules (20). For both batches of liposomes, GM3 inclusion resulted in a higher (~20-fold) liposome uptake when compared to non-GM3-containing counterparts (gating according to **Supplementary Figure 2A**). As expected, pretreatment with a blocking antibody specific for CD169 abrogated the enhanced uptake of GM3 liposomes to background levels (**Figures 1B, D**). This reduction in uptake was not observed when we used an isotype control antibody (**Supplementary Figure 1D**). Unexpectedly we also observed some binding of TLR4L/MDP liposomes to moDC that was not blocked by the CD169 blocking antibody (**Figure 1D**).

Subsequently, we determined the ability of adjuvant-containing GM3 liposomes to mature moDC. MoDC were either non-treated or treated for 15 minutes with a blocking antibody specific for CD169, prior to liposome incubation. Unbound liposomes were washed away and moDC were cultured overnight and stained for maturation markers. While GM3 liposomes without adjuvant (control) were not able to induce maturation (data not shown), GM3 liposomes harboring the ligands of TLR4 or 7/8 were able to induce higher levels of the maturation marker CD86 (**Figures 1E, F**). While the oxidized lipid PGPC was not able to upregulate maturation, MDP-containing GM3 liposomes did mature moDC (**Figures 1E, F**, respectively). We observed that the maturation induced by TLR4L was efficiently inhibited by the CD169-blocking antibody, but this was not observed for maturation induced by liposomes containing TLR7/8L and/or MDP (**Figures 1E, F**). In addition, we observed that TLR4L-containing GM3 liposomes induced IL-1β expression and secretion and that adjuvant-containing GM3 liposomes were well tolerated, since excessive cell death upon stimulation was not observed (**Supplementary Figures 1E–H**). These results indicate that GM3 liposomes containing the TLR4L MPLA are more potent inducers of moDC maturation, when compared to GM3 liposomes containing TLR7/8L (3M-052) and/or MDP. We did not observe an effect on maturation upon PGPC incorporation in GM3 liposomes and PGPC or MDP in combination with TLRL did not result in higher maturation.

### GM3 Liposomes With Adjuvant Are Efficiently Taken Up by Mouse Splenic CD169-Macrophages and MPLA Inclusion Induces Robust DC Maturation

**Figure 2:**
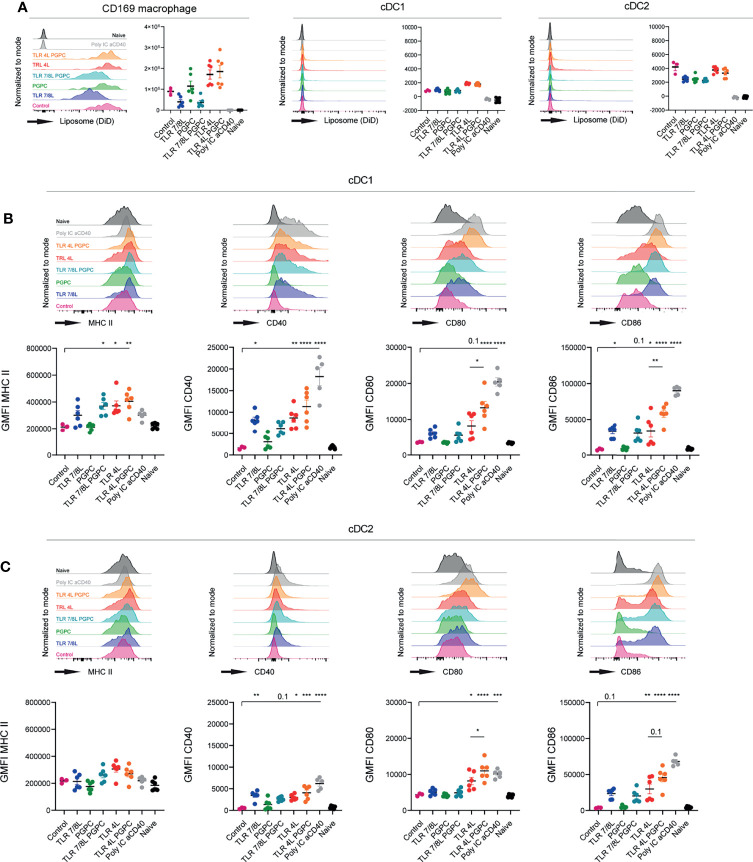
TLRL-containing GM3 liposomes induce DC maturation *in vivo*, but additional incorporation of PGPC has minor effects. **(A–C)** Mice were i.v. injected with different liposomal formulations, poly IC and aCD40 antibody as positive control, or left uninjected (naive). 16h after injection, spleens were collected and a single cell suspension was used for flow cytometry staining and analysis. Indicated are representative histograms and the quantified DiD-signal as average ± SEM, [*n* = 6 (*n* = 3 for control liposome)], data pooled from two independent experiments **(A)**. Indicated are representative histogram overlays and quantified average ± SEM of maturation markers on cDC1 and cDC2 [**(B, C)**, respectively) [*n* = 6 (*n* = 3 for control liposome)], data pooled from two independent experiments. *p < 0.05, **p <0.01, ***p < 0.005 and ****p < 0.0001.

Our previous studies have extensively demonstrated that GM3 liposomes bind to mouse splenic CD169^+^ macrophages, which in collaboration with cDC1 activate T cell responses (48, 49, 60). In order to determine whether adjuvant-containing GM3 liposomes also effectively target to CD169^+^ macrophages and subsequently mature cDC1 and cDC2 we performed both *in vitro* as well as *in vivo* experiments that provided similar results [*in vitro* uptake and maturation (**Supplementary Figure 3**)]. In addition, we evaluated soluble adjuvants next to adjuvant-containing GM3 liposomes. The maturation induced by liposomal TLR4L was stronger compared to an equal soluble dose of TLR4L (**Supplementary Figure 4A**). Furthermore, both unformulated and GM3 liposome incorporated TLR7/8L induced maturation of DCs *in vitro*, albeit that maturation was variable and dependent on the readout (i.e. CD80 or CD86) (**Supplementary Figure 4B**). In contrast, PGPC or GM3 liposome incorporated PGPC did not induce DC maturation *in vitro* (**Supplementary Figure 4B**). To determine *in vivo* uptake, we immunized C57BL/6 mice intravenously (i.v.) with GM3 liposomes containing adjuvants. We observed that 16h after i.v. immunization, all GM3 liposomes were effectively taken up by splenic CD169^+^ macrophages. Some uptake by conventional DC1 and DC2 was also observed, but these levels were extremely low compared to uptake by CD169^+^ macrophages (**Figure 2A**). Thus, adjuvant-containing GM3 liposomes effectively target CD169^+^ macrophages *in vivo*.

Next, we evaluated to what extent GM3 liposomes were able to mature cDC1 and cDC2 *in vivo*. GM3 liposomes that contained TLR4L were found to be highly effective in the maturation of cDC1 and cDC2 (**Figures 2B, C**). Interestingly, adding PGPC to liposomal TLR4L, increased the expression of CD80 and CD86 on cDC1 and cDC2, but did not affect the level of MHC class II and CD40 expression. This combined effect of PGPC plus TLR4L was observed in two independent experiments with two independent batches of liposomes [characteristics in (**Supplementary Figure 5A**)]. Immunization with GM3 liposomes containing TLR7/8L also resulted in DC maturation, but to a lower degree when compared to TLR4L. The addition of PGPC to TLR7/8L did not further enhance DC maturation (**Figures 2B, C**). Sole incorporation of PGPC did not elicit an effect as the level of maturation markers was similar to that of control GM3 liposomes immunized, and that of non-injected mice. Thus, TLR4L-containing GM3 liposomes efficiently mature DC *in vivo* and additional incorporation of PGPC increases the expression of CD80/86 on cDC1 and cDC2.

**Figure 3:**
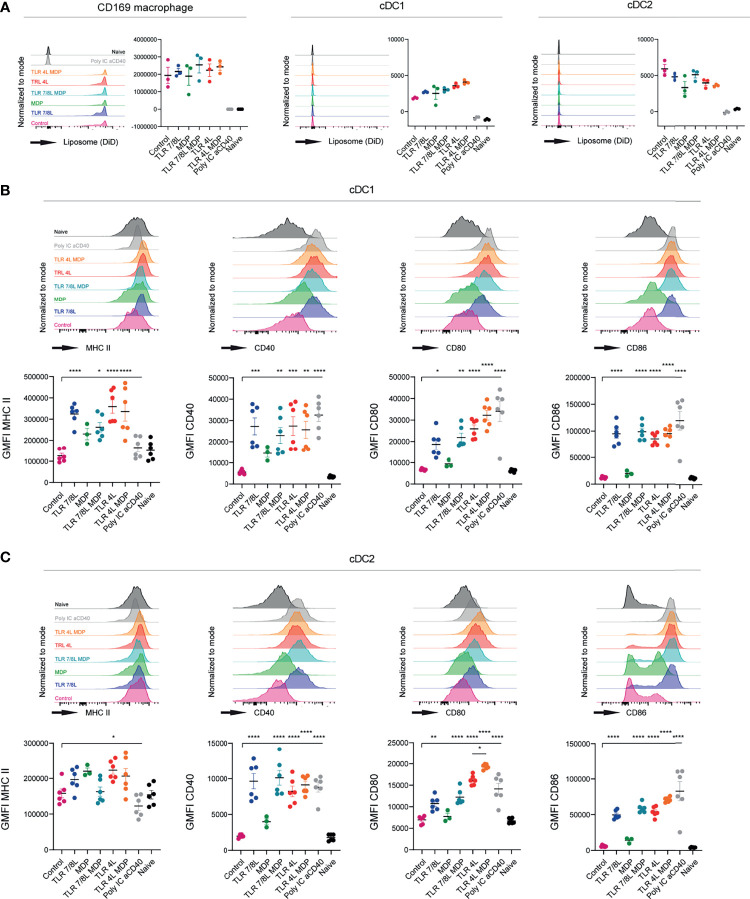
TLRL-containing GM3 liposomes induce DC maturation *in vivo* and additional incorporation of MDP has no effect. **(A–C)** Mice were i.v. injected with different liposomal formulations, poly IC and aCD40 antibody as positive control, or left uninjected (naive). 16h after injection, spleens were collected and a single cell suspension was used for flow cytometry staining and analysis. Indicated are representative histogram overlays and the quantified DiD-signal as average ± SEM (*n* = 3), representative of two independent experiments **(A)**. Indicated are representative histogram overlays and quantified average ± SEM of maturation markers on cDC1 and cDC2 [**(B, C)**, respectively] (*n* = 3-6), data pooled from two independent experiments. *p < 0.05, **p <0.01, ***p < 0.005 and ****p < 0.0001.

Subsequently, we performed a similar experiment with GM3 liposomes containing MDP, alone or in combination with TLR4 or TLR7/8 ligands. All GM3 liposomes containing adjuvant were found to be taken up by CD169-expressing macrophages, and to a significantly lower extent by cDC1 and cDC2 (**Figure 3A**). Again, we observed efficient maturation of cDC1 and cDC2 (based on MHC II, CD40 and CD80/86, or CD40 and CD80/86, respectively) when a TLRL was present (**Figures 3B, C**). While soluble or liposomal MDP induced maturation of cDC2 *in vitro* (**Supplementary Figure 4B**), sole inclusion of MDP tended to upregulate MHC II and CD40 *in vivo*, but did not affect CD80/86 and its overall effect was minor. This was observed with two independent batches in separate experiments (characteristics in **Supplementary Figure 5B**). In conclusion, incorporation of TLR4L (MPLA) in GM3 liposomes was most potent in inducing DC maturation, while TLRL7/8L (3M-052) was less potent. Addition of the inflammasome activators PGPC or MDP to TLRL containing GM3 liposomes resulted in only minor additional effects on maturation.

### Immunization With TLR4L-Containing GM3 Liposomes Induces Stronger T Cell Responses Compared to Immunization With TLR7/8L-Containing GM3 Liposomes

**Figure 4:**
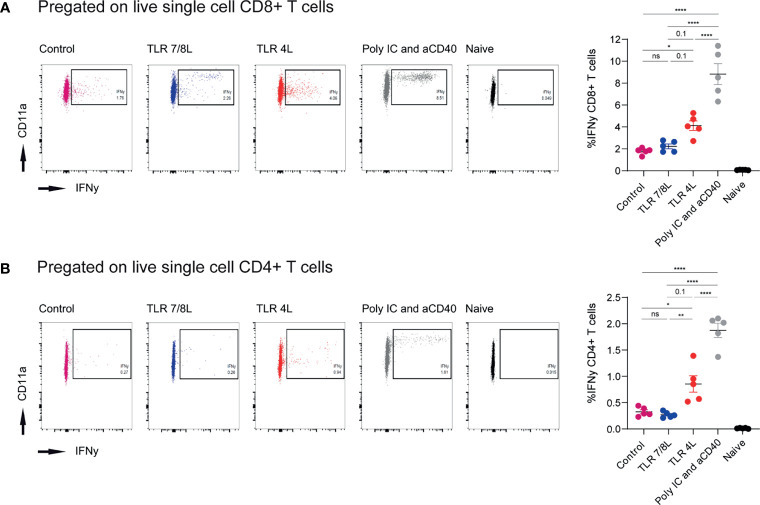
Immunization with antigen-containing GM3 liposomes and TLR4L-containing GM3 liposomes elicits more robust antigen-specific immune responses than antigen-containing GM3 liposomes and TLR7/8L-containing GM3 liposomes. **(A, B)** Mice were i.v. immunized with antigen-containing GM3 liposomes (22.5 nmol phospholipid) that were supplemented with control GM3 liposomes (no TLR ligand), TLR7/8L-containing GM3 liposomes, TLR4L-containing GM3 liposomes (200 nmol phospholipid), or soluble poly IC and aCD40 antibody as positive control. After 7 days, spleens were collected and a single cell suspension was used for peptide restimulation for 5 or 25 hours for CD8^+^ and CD4^+^ T cells, respectively. Indicated are the representative dot plots of IFNγ production after peptide restimulation and the average percentage ± SEM of IFNγ-producing CD8^+^ and CD4^+^ T cells [**(A, B)**, respectively] (*n* = 3-5). **p < 0.01 and ****p < 0.0001. *p < 0.05, ns, not significant.

Our next aim was to determine the capacity of the different liposomes to stimulate polyclonal T cell responses. Hence, adjuvant-containing GM3 liposomes were mixed with antigen-containing GM3 liposomes (in a 1:1 ratio). While the former liposomes were described above, the latter antigen-containing GM3 liposomes were described previously (49). Briefly, these GM3 liposomes had a mean size of 224 ± 15.5 nm, a PDI of 0.2 ± 0.05, a mean zeta-potential of -44.5 mV and contained a synthetic long peptide derived from the model antigen ovalbumin (OVA_247-279_). This peptide is immunogenic and contains both a CD4- and CD8-T cell epitope (OVA_262-276_ and OVA_257-264_, respectively). Mixed liposomes or antigen-containing GM3 liposomes supplemented with soluble (i.e. not included in liposomes) adjuvant (aCD40 and poly IC) were administered i.v. to C57BL/6 mice. After 7 days, we analyzed the splenic compartment for the presence of antigen-specific T cells by intracellular IFNγ staining after peptide restimulation (gating according to **Supplementary Figure 2C**). While immunization with GM3 liposomes containing TLR4L tended to result in stronger responses as compared to the GM3 liposomes with TLR7/8L, this was not significant. The highest CD8^+^ and CD4^+^ responses were observed when we co-injected a strong soluble adjuvant as stimulus (**Supplementary Figures 6A, B**). As the overall responses in this experiment were relatively minor, we also mixed adjuvant- and antigen-containing GM3 liposomes in a 9:1 ratio. In addition, we included a control GM3 liposome that lacked a TLR ligand. Immunization with GM3 liposomes containing the ligand for TLR4 elicited more potent T cell responses as compared to GM3 liposomes with the ligand for TLR7/8, or control GM3 liposomes. In fact, TLR4L-containing GM3 liposomes were able to induce more robust T cell responses as compared to the control GM3 liposome, but TLR7/8L-containing GM3 liposomes failed to augment the response. The clear response elicited by TLR4L-containing GM3 liposomes was consistent with a more robust maturation observed with TLR4L-containing GM3 liposomes. However, the T cell responses induced by TLRL-containing GM3 liposomes were significantly lower than the response induced by the addition of a strong soluble adjuvant next to antigen-containing GM3 liposomes (**Figures 4A, B**).

### Addition of Inflammasome Ligands to TLR4L-Containing GM3 Liposomes Does Not Enhance Its Potency

**Figure 5:**
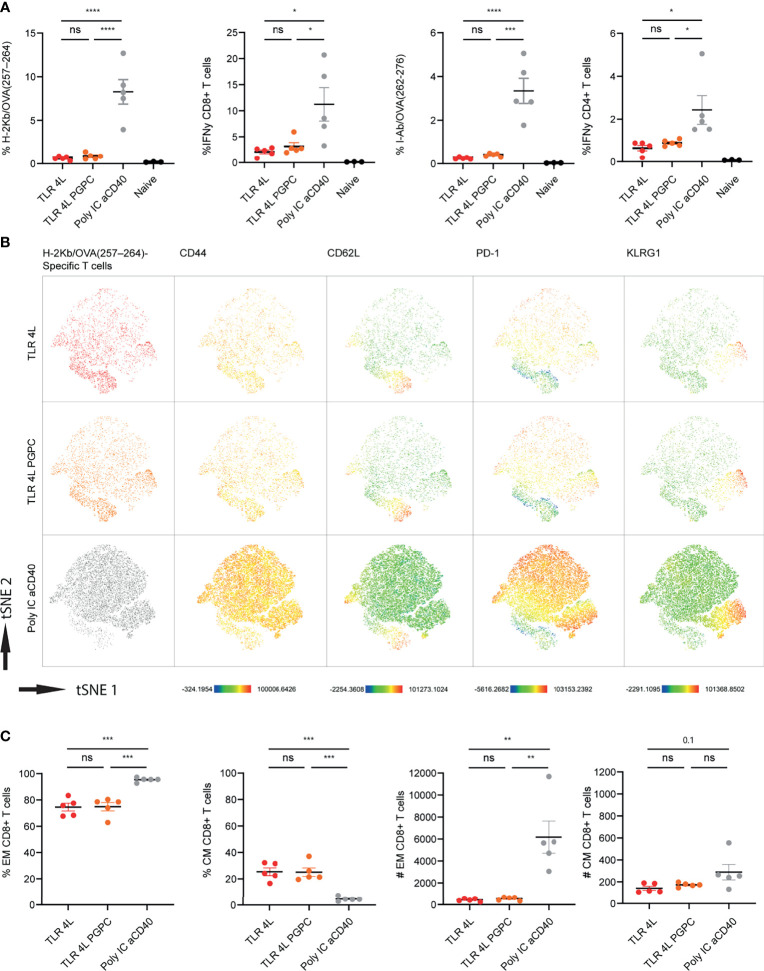
Addition of PGPC does not augment the potency of TLR4L-containing GM3 liposomes. **(A)** Mice were i.v. injected with different liposome formulations, or antigen-containing liposomes with poly IC and aCD40 antibody as soluble adjuvant as a positive control, or left uninjected (naive). 7 days after injection, spleens were collected and a single cell suspension was used for tetramer staining or peptide restimulation. Indicated is the average ± SEM of the percentage of IFNγ−producing CD8^+^ and CD4^+^ T cells upon peptide restimulation and CD8^+^ and CD4^+^ tetramer binding cells (*n* = 3-5). **(B)** H-2Kb/OVA_257-264_ binding T cells as identified in A were clustered using high-dimensional data reduction. H-2Kb/OVA_257-264_ binding T cells are indicated (groups were indicated with different colors in the first column), as well as their expression levels of CD44, CD62L, PD-1 and KLRG1. **(C)** H-2Kb/OVA_257-264_ binding T cells were subdivided in CD44^+^ Tem and CD44^+^CD62L^+^ Tcm cells. Indicated is the average ± SEM of the percentage (of antigen-specific CD8^+^ T cells) and number of cells identified as Tem or Tcm (based on 10^6^ events) (*n* = 5). *p < 0.05, **p <0.01, ***p < 0.005 and ****p < 0.0001. ns, not significant.

Immunization with antigen-containing liposomes supplemented with control or TLR7/8L-containing liposomes induced only low T cell responses. Thus, we continued with TLR4L-containing GM3 liposomes to assess the potential additional effect of the oxidized lipid PGPC and MDP on the T cell responses. Here, we incorporated the long synthetic OVA_247-279_ peptide in the adjuvant-containing GM3 liposomes, that were similar in size, PDI and zeta-potential as described above (**Supplementary Figure 5A**). One week after i.v. immunization, we analyzed antigen-specific CD4^+^ and CD8^+^ T cell responses by intracellular cytokine staining after peptide restimulation and tetramer staining (gating according to **Supplementary Figures 2C, D**, respectively). While we detected T cell responses after immunization with TLR4L-containing GM3 liposomes, the magnitude of the immune response, for both CD8^+^ and CD4^+^ T cells was lower when compared to the control group which we immunized with a strong soluble adjuvant (**Figure 5A**). In addition, we performed a high dimensionality reduction *t*-distributed stochastic neighbor embedding (tSNE) analysis on H-2K^b^/OVA_257–264_ specific CD8^+^ T cells and subsequently subdivided previously concatenated populations into the different groups for unbiased phenotyping. Since immunization with a strong soluble adjuvant induced the strongest response, most events (antigen-specific CD8^+^ T cells) were present in this group (**Figure 5B**). Furthermore, we observed that immunization with TLR4L-containing GM3 liposomes with or without PGPC resulted in a high abundance of CD44^+^ CD62L^-^ effector memory T cells (Tem) and a population of CD44^+^ CD62L^+^ central memory T cells (Tcm), of which the latter was higher in percentage as compared to the control with aCD40 and poly IC as strong soluble adjuvant, but similar in numbers (**Figures 5B, C**) [based on 10^6^ total events, the total number of splenocytes was similar between immunized groups (data not shown)]. The altered distribution between Tem and Tcm, but similar numbers of Tcm cells were also detected when we analyzed antigen-specific CD4^+^ T cells (**Supplementary Figure 7B**). Furthermore, immunization with a strong soluble adjuvant induced high expression of PD-1 on the Tem, while this was lower when we immunized with MPLA or MPLA/PGPC-containing GM3 liposomes (**Figure 5B** and **Supplementary Figure 7A**). We also detected a population of KLRG1-expressing Tem cells in all liposomal immunization strategies (**Figure 5B** and **Supplementary Figure 7A**). The additional incorporation of the inflammasome ligand PGPC in TLR4L-containing GM3 liposomes did not contribute to differences in the phenotype of antigen-specific T cells when compared to TLR4L-containing GM3 liposomes.

**Figure 6:**
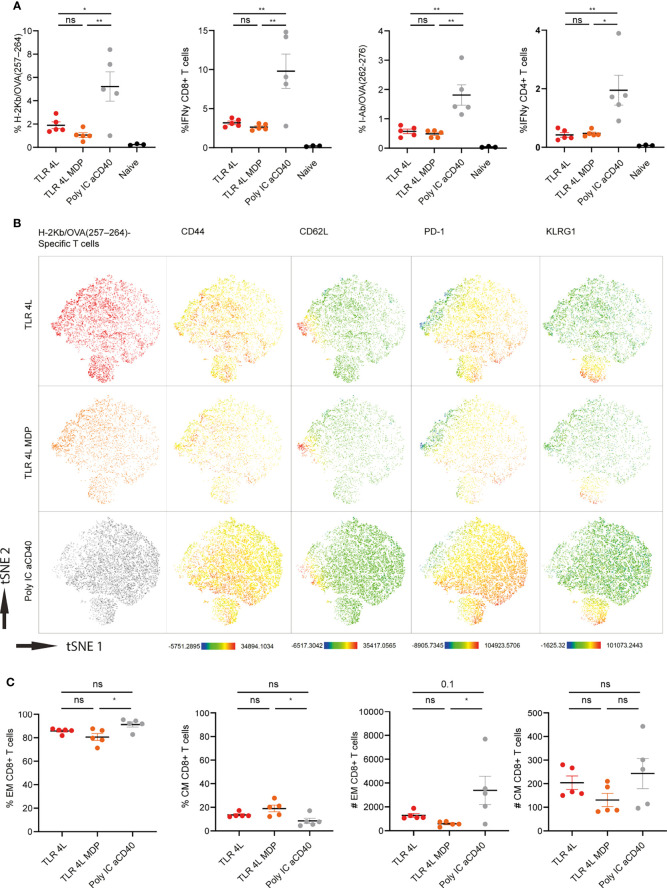
Addition of MDP does not augment the potency of TLR4L-containing GM3 liposomes. **(A)** Mice were i.v. injected with different liposome formulations, or antigen-containing liposomes with poly IC and aCD40 antibody as soluble adjuvant as a positive control, or left uninjected (naive). 7 days after injection, spleens were collected and a single cell suspension was used for tetramer staining or peptide restimulation. Indicated is the average ± SEM of the percentage of IFNγ−producing CD8^+^ and CD4^+^ T cells upon peptide restimulation and CD8^+^ and CD4^+^ tetramer binding cells (*n* = 3-5). **(B)** H-2Kb/OVA_257-264_ binding T cells as identified in A were clustered using high-dimensional data reduction. H-2Kb/OVA_257-264_ binding T cells are indicated phenotyping (groups were indicated with different colors in the first column) as well as their expression levels of CD44, CD62L, PD-1 and KLRG1. **(C)** H-2Kb/OVA_257-264_ binding T cells were subdivided in CD44^+^ Tem and CD44^+^CD62L^+^ Tcm cells. Indicated is the average ± SEM of the percentage (of antigen-specific CD8^+^ T cells) and number of cells identified as Tem or Tcm (based on 10^6^ events) (*n* = 5). *p < 0.05 and **p <0.01. ns, not significant.

Next, we performed a similar experiment with TLR4L-containing GM3 liposomes with or without MDP. Consistent with our previous finding, TLR4L-containing GM3 liposomes induced antigen-specific T cell response, albeit lower when compared to our positive control (**Figure 6A**). The addition of the inflammasome stimuli MDP to TLR4L-containing GM3 liposomes did not augment the effect that was elicited by TLR4L-containing GM3 liposomes (**Figure 6A**). Furthermore, we performed a high dimensional data reduction on H-2K^b^/OVA_257–264_ specific CD8^+^ T cells and subsequently analyzed the phenotype of these antigen-specific cells. We detected a robust induction of Tem cells upon immunization with a strong soluble adjuvant, that was less pronounced when we immunized with liposomal adjuvant (TLR4L with or without MDP), whereas the Tcm response differed less between the groups (**Figures 6B, C**) [based on 10^6^ total events, total number of splenocytes was similar in the immunized groups (data not shown)]. Again, this was also reflected by Tem and Tcm distribution within the antigen-specific CD4^+^ T cell compartment (**Supplementary Figure 8B**). In addition, immunization with a strong soluble adjuvant, aCD40 and poly IC, induced high levels of PD-1 on antigen-specific CD8^+^ Tem cells. All vaccination strategies resulted in the presence of KLRG1-expressing Tem cells (**Figure 6B** and **Supplementary Figure 8A**). Thus, immunization with antigen and TLR4L-containing GM3 liposomes does translate into T cell responses, but robust expansion of Tem cells, as observed with a strong soluble adjuvant, was not detected.

In conclusion, TLRL delivery in GM3 liposomes results in maturation of both cDC1 and cDC2 and the activation of T cell responses. The additional inclusion of oxidized lipid PGPC or MDP had minor effects on DC maturation, and did not further enhance T cell priming. Our data indicate that adjuvant inclusion in GM3 liposomes is a viable strategy to induce DC maturation and T cell responses. Further studies will focus on different (combinations of) liposomal adjuvants to optimize T cell responses.

## Discussion

In this work, we build upon our previous observations that GM3-targeted liposomes are effectively taken up by CD169-expressing human and mouse cells (48, 49, 58, 59), and that antigen-containing GM3 liposomes together with a strong soluble adjuvant (aCD40 and poly IC) can elicit potent immune activation and anti-tumor responses (48, 49). In our current study we aimed to utilize the versatile nature of liposomes to further augment the potency of liposomal cancer vaccination by inclusion of lipophilic adjuvants, which would remove the need for systemic administration of adjuvant. We incorporated ligands of TLR7/8 (3M-052) and TLR4 (MPLA) in GM3 liposomes and tested their potency alone, and in combination with inflammasome stimuli, the oxidized lipid PGPC or the NOD2 ligand MDP, to synergistically induce cellular maturation and subsequent T cell priming.

We detected robust human moDC maturation when TLR4L was incorporated into GM3 liposomes. The maturation by MPLA-containing GM3 liposomes was dependent on the presence of GM3 and binding to CD169. MPLA-containing GM3 liposomes also led to maturation of murine DCs and were more potent than a soluble dose of MPLA. In contrast, GM3 liposomes containing TLR7/8L were less stimulatory and blocking of CD169 did not inhibit maturation induced by TLR7/8L or MDP liposomes as effective. Indeed, while the TLR7/8L in this study contains hydrophobic regions that allow for its incorporation in the liposomal bilayers (61), this ligand may not be as stably embedded in the bilayer as MPLA that contains six acyl chains. Similarly, the NOD2 ligand used in this study contains a single lipid tail (62), which may indicate a relatively weak interaction with the liposomal bilayers and potential release from the liposomes. Our data are very similar to those obtained by Boks and coworkers which targeted adjuvant-containing Lewis X-liposomes to DC-SIGN on moDC (16). In this study, maturation induced by TLR4L-containing Lewis X-liposomes was also dependent on the presence of targeting moieties and could be blocked with a blocking DC-SIGN antibody. Maturation induced by TLR7/8L-containing Lewis X-liposomes was less dependent on Lewis X mediated targeting and induced maturation was less sensitive to a blocking antibody (16). These data together with ours indicate that the combination of TLR4L and a targeting ligand leads to the most optimal DC maturation and appears to be a valid approach to augment the potency of liposomal cancer vaccines.

We additionally included the oxidized lipid PGPC in our liposomal formulations to aim for a synergistic or additive effect on DC maturation and subsequent T cell responses. While synergistic effects of oxidized lipids next to TLR4L stimulation was previously observed (52), we failed to see strong effects. There are a few important considerations that may explain this difference. First, while Zhivaki and coworkers temporally spaced their stimuli and first primed with a TLR4L followed by PGPC, we incorporated both TLR4L and PGPC in the same particles. Furthermore, other studies also described suppressing effects of oxidized lipids. Pretreatment of cells with oxidized lipids reduced subsequent LPS-mediated maturation (63) and simultaneous treatment with certain species of oxidized lipids with LPS diminished LPS-induced cytokine secretion (64). Thus, the tissue context and timing of exposure to TLR ligands/oxidized lipids appears to be an important determinant of subsequent cytokine secretion and cellular maturation. Second, the effect of oxidized lipids may also depend on the recipient cell type, as DC generated with granulocyte–macrophage colony-stimulating factor (GM-CSF) were more sensitive to oxidized lipids as compared to conventional fms-like tyrosine kinase 3 ligand-dependent DC (54). More detailed, the bone marrow-derived DC model consist of a mixed population of monocyte-derived macrophages and DC and inflammasome ligands elicited more robust responses in GM-CSF-derived macrophages than GM-CSF-derived DC (65). The requirement of CD14 expression to elicit an effect of oxidized lipid may explain differences observed between the heterogeneous mixture of cells generated from the bone marrow with GM-CSF and conventional (splenic) DC (66). In addition, expression of IRF8 and IRF4, by cDC1 and cDC2, respectively, was associated with reduced inflammasome activation (67). Thus, targeting of liposomal vaccine components to monocyte-derived cells may result in stronger inflammasome-mediated responses as compared to those elicited by targeting to conventional DC. Third, as we aimed to administer the oxidized lipids in liposome-embedded form we were limited with regard to the dose of PGPC. While we started with a dose of 2 mol% of liposomal PGPC, we later also tested a higher dose of 5 mol%. However, this did not result in stronger effects as compared to those elicited by 2 mol% liposomal PGPC (data not shown). Incorporation of an even higher dose of PGPC in liposomes may result in micelle formation (68). Therefore, we did not attempt to prepare liposomes containing a higher amount of PGPC that would enable the administration of the same dose of PGPC, as used by others (52). While liposomal delivery of cargo was expected to augment immunological effects compared to the administration in non-liposomal form, we observed only minor effects on maturation caused by the liposomal oxidized lipid (combined with TLR4L) and in addition, this did not translate to enhanced T cell responses. Therefore, the potential beneficial use of PGPC in liposomal cancer vaccine immunization requires further investigation of mechanisms of delivery to find conditions in which stronger synergistic effects can be achieved.

There is a long-standing interest in liposomal muramyl (tri)peptides for inducing immune activation (69, 70). Recently, renewed interest in MDP sparked, as it was shown to induce a state of DC hyperactivity, when combined with TLR ligands (55, 56). Indeed, stimulation of human cDC2 at different time points with TLR7/8L and MDP led to potent cytokine secretion *in vitro* (56). In addition, incubation with nanoparticles containing MDP and TLR7/8L induced additive murine DC maturation *in vitro.* While the effect on bone marrow-derived DC was robust, the effect on primary isolated splenic DC was less pronounced (55). We detected responses to liposomal TLR7/8 ligand stimulation *in vitro*, but failed to see a robust additive effect of liposomal MDP *in vitro* and *in vivo*. Indeed, this may be a consequence of the cell types we studied, since, similar as discussed above for PGPC, bone marrow-derived DC (stimulated with GM-CSF) were better responders as compared to primary splenic conventional DC (55). While incorporation of MDP alone elicited some effect on DC maturation, this effect was not as potent as in the case of liposomal TLR ligands. Further research should focus on temporally spaced administration of these different liposomes in different concentrations to optimize synergistic DC maturation and subsequent T cell responses.

Next to DC maturation, we aimed to assess to what extent matured DC prime T cells upon liposomal immunization. We previously used liposomes together with soluble poly IC and activating anti-CD40 antibody as adjuvant for immunization (48, 49). In order to search for immunization strategies with less systemic side effects, we assessed various liposomal adjuvants and took along poly IC and aCD40 antibody as positive control. While immunization with antigen and TLRL-containing GM3 liposomes did translate into T cell responses, the magnitude was lower as compared to poly IC and aCD40 antibody administrated as soluble adjuvants. In addition, we detected a robust expansion of Tem cells upon immunization with GM3 liposomes supplemented with a strong soluble adjuvant that was not observed when we immunized with TLR4L-containing GM3 liposomes. Remarkably, the numbers of Tcm cells after immunization with GM3 liposomes supplemented with a aCD40 and poly IC were similar to those induced by TLR4L-containing GM3 liposomes. This observation is based on the relatively simple distinction between antigen-specific Tem cells (CD44^+^) and Tcm cells (CD44^+^CD62L^+^), thus limiting the possible appreciation of Tcm cell heterogeneity (71). The formation of Tcm cells was described to depend on the duration of antigenic stimuli, and less dependent on the inflammatory status of the immune system (72). In addition, Tcm exhibited a lower proliferative index as compared to Tem (72). Consistent with these observations, we observed that the absolute numbers of CD8^+^ and CD4^+^ Tcm cells were comparable when we immunized with GM3 liposomes supplemented with soluble aCD40 and poly IC or TLR4L-containing GM3 liposomes. While the inflammatory status of the immune system after immunization with liposomal TLRL will likely be lower when compared to soluble poly IC and aCD40 antibody, antigen presentation is expected to be similar, explaining the similar numbers of Tcm observed in our immunization strategy. Further research should elucidate whether the use of different types and combinations of adjuvant induce differences in memory responses after a second boost of antigen.

In conclusion, our study demonstrates that GM3 liposomes can be used for co-delivery of adjuvant and antigen. TLR4L-containing GM3 liposomes are able to mature DCs and induce CD4^+^ and CD8^+^ T cell priming. Further research into different combinations of adjuvants incorporated in GM3 liposomes will be necessary to achieve optimal effects upon immunization. Thus far, our results clearly support the development of liposomal vaccination platforms to stimulate anti-cancer immune responses.

## Data Availability Statement

The raw data supporting the conclusions of this article will be made available by the authors, without undue reservation.

## Ethics Statement

The animal study was reviewed and approved by National Committee for Animal Experiments, Vrije Universiteit, Amsterdam UMC.

## Author Contributions

The project conceptualization was performed by MN, LC, GS, and JH. MN, LC, RB, KO, JG, AW, AA, and SB carried out the experiments, that were analyzed by MN. Materials were provided by HK and supervision provided by CN, YK, GS, and JH. The initial manuscript draft was prepared by MN and JH, remaining authors reviewed and edited the manuscript. All authors contributed to the article and approved the submitted version.

## Funding

This work was supported by grants from NWO ZonMW (TOP 91218024) to JH and GS, grants from the Dutch Cancer Society (VU2016-10449), from the Phospholipid Research Center (JDH-2020-082/1-1), from Health Holland TKI-PPP to JH and a grant from the NUS School of Medicine Nanomedicine Research Programme (NUHSRO/2021/034/TRP/09/Nanomedicine) to GS.

## Conflict of Interest

The authors declare that the research was conducted in the absence of any commercial or financial relationships that could be construed as a potential conflict of interest.

## Publisher’s Note

All claims expressed in this article are solely those of the authors and do not necessarily represent those of their affiliated organizations, or those of the publisher, the editors and the reviewers. Any product that may be evaluated in this article, or claim that may be made by its manufacturer, is not guaranteed or endorsed by the publisher.
