# Supplementary material for: Incorporation of Toll-Like Receptor Ligands and Inflammasome Stimuli in GM3 Liposomes to Induce Dendritic Cell Maturation and T Cell Responses
Source: Front Immunol. 2022 Feb 18;13:842241. doi: 10.3389/fimmu.2022.842241 (PMC8895246; doi:10.3389/fimmu.2022.842241)
Supplement: Supplementary file 1 [file DataSheet_1.docx]

Supplementary Material

**
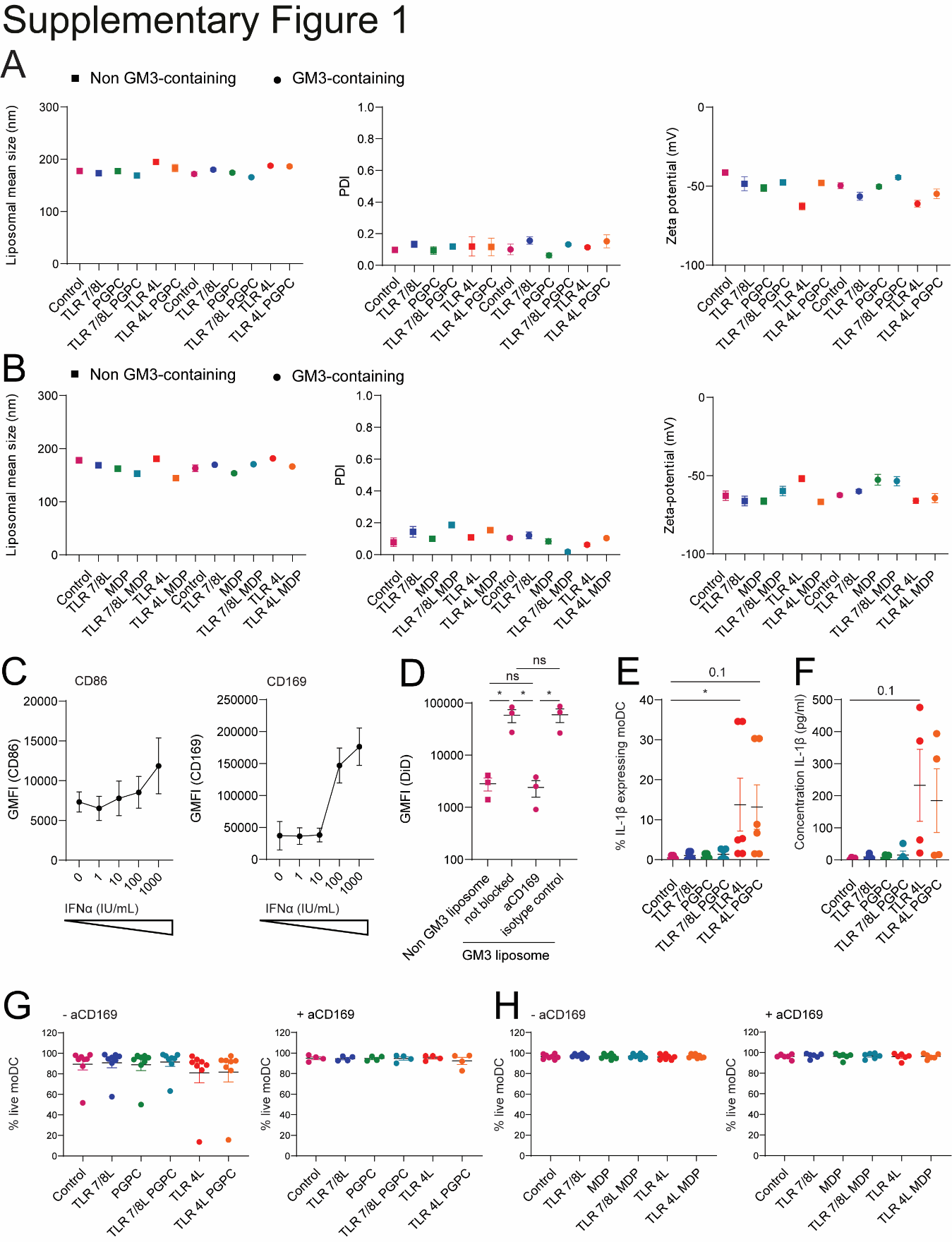
**

**Supplementary Figure 1:** **Adjuvant inclusion in liposomes does not alter physicochemical properties of liposomes, does not lead to extensive cell death, TLR 4L inclusion induces IL-1β expression and low dose IFN-treatment induces CD169, but not CD86 expression on moDC**. A/B) TLRL and/or PGPC or MDP-containing (GM3) liposomes were characterized by measurement of the liposomal mean size, PDI and zeta-potential, indicated is the average ± SEM of a technical triplicate. C) MoDC were plated and non-treated or treated with various doses of interferon α (IFNα) for 2 days. Subsequently, cells were stained and the expression of CD86 and CD169 was determined using flow cytometry. Indicated is the average expression of CD86 or CD169 ± SEM (*n* = 3-4). D) MoDC were non-treated or incubated with a blocking antibody or isotype control (anti-Langerin) for 15 minutes and subsequently incubated with a non-GM3-containing liposome or GM3-containing liposomes for 45 minutes at 37 °C. Next, unbound liposomes were washed away and liposomal DiD signal was quantified using flow cytometry. Indicated is the average DiD-signal ± SEM (*n* = 3). E) MoDC were incubated with GM3 liposomes for 2 hours at 37 °C, after extensive washing cells were incubated for an additional 3 hours in the presence of Golgiplug. The cells were permeabilized and stained for IL-1β and the percentage of IL-1β expressing cells was determined using flow cytometry analysis. Indicated is the average percentage of IL-1β expressing cells ± SEM (*n* = 6). F) MoDC were incubated with liposomes for 45 minutes at 37 °C. Next, unbound liposomes were washed away and cells were incubated for 15-18h at 37 °C, after which the supernatant was collected and the concentration of IL-1β determined using an ELISA-based assay. Indicated is the average concentration of IL-1β ± SEM (*n* = 4). G) MoDC were non-treated or incubated with a blocking antibody for 15 minutes and subsequently incubated with liposomes for 45 minutes at 37 °C. Next, unbound liposomes were washed away and cells were incubated for 15-18h at 37 °C, after which the cells were stained and the percentage of live cells was determined by flow cytometry analysis. Indicated is the average percentage of live cells ± SEM (*n* = 4-8). H) as in G, but cells were pretreated with a low dose of IFNα (100 IU/mL) (*n* = 6). * p < 0.05.

**
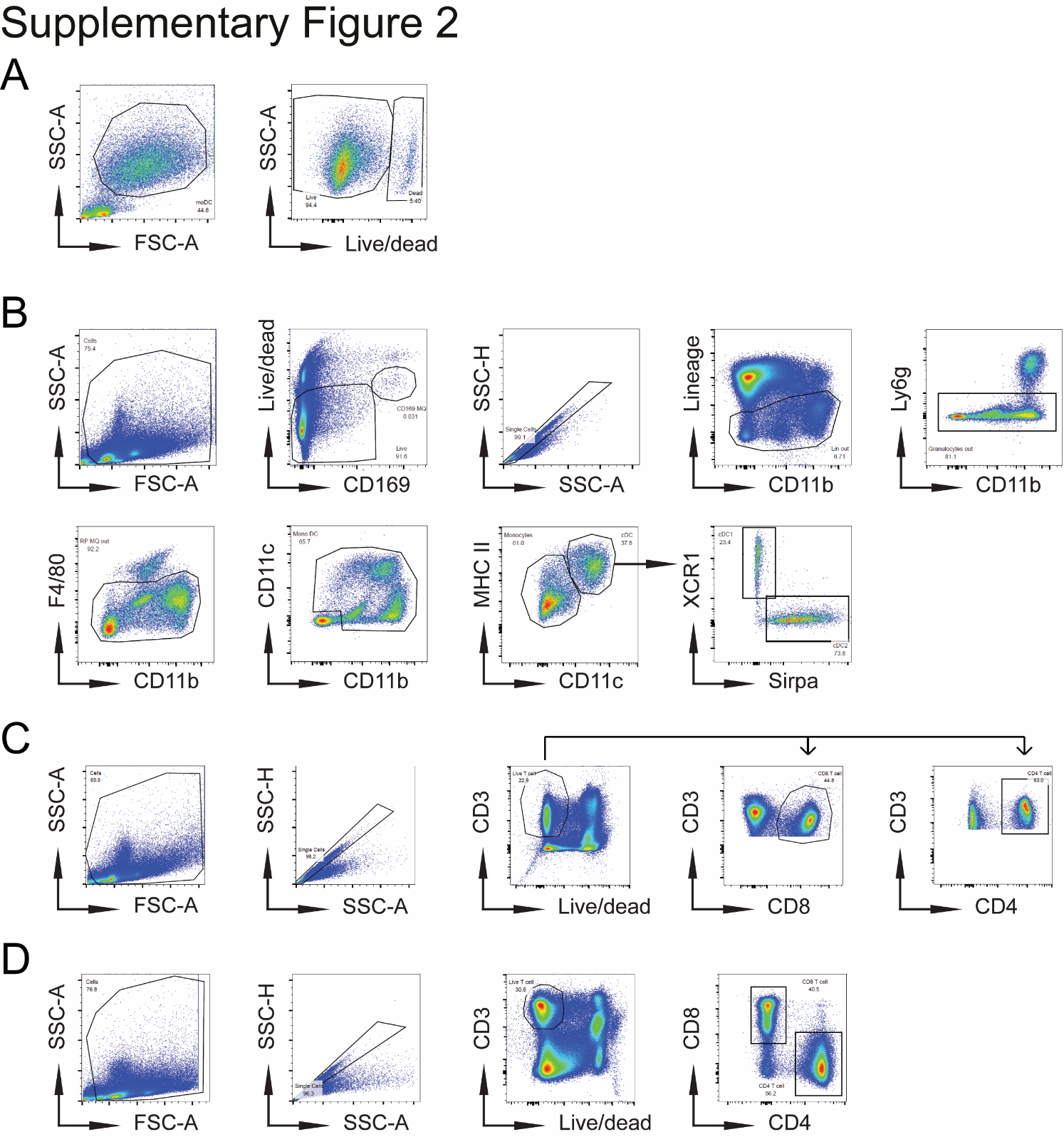
**

**Supplementary Figure 2: Gating strategy to identify human moDC, murine splenic DC or T cells.** A) moDC were identified and separated from debris and contaminating lymphocytes based on their scatter profile, subsequently live cells were gated. B) After gating out debris, murine CD169^+^ macrophages were identified based on CD169 expression. Subsequently, single cells were selected and lineage cells (CD3/CD19/CD56) were gated out, followed by Ly6g^+^ granulocytes and F4/80^+^ red pulp macrophages. CD11b/CD11c positive cells were then selected to identify MHC II and CD11c double positive cDC, that were subdivided in XCR1^+^ cDC1 and Sirpα^+^ cDC2. C/D) T cells were identified after gating out debris, selecting single cells and live CD3^+^ cells. Subsequently, CD8^+^ or CD4^+^ T cells were gated.

**
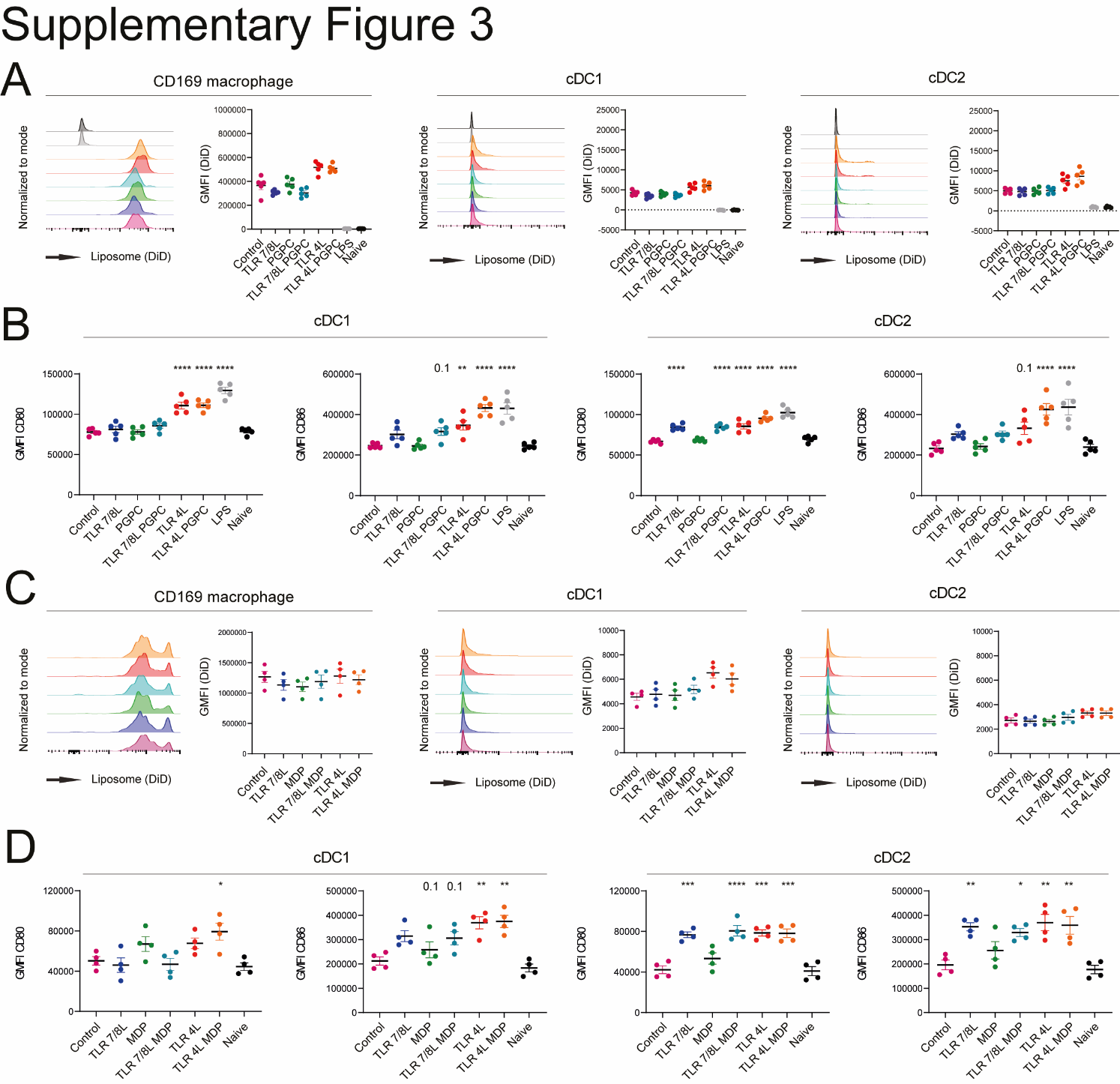
**

**Supplementary Figure 3: GM3 liposomes are taken up by CD169-expressing macrophages and additional TLR4L inclusion induces the most robust DC maturation in vitro.** A/C) A single cell suspension of murine splenocytes was incubated with liposomes (100 uM phospholipid) for 45 minutes at 37 °C. Subsequently, cells were washed, stained and liposome uptake determined using flow cytometry analysis. Liposome uptake by splenic CD169-expressing macrophages, cDC1 and cDC2 is illustrated and indicated is the average DiD-signal ± SEM (*n* = 5). B/D) Splenocytes were treated as in A, but after an initial 45 minutes uptake at 37 °C and extensive washes, cells were incubated overnight at 37 °C to mature. Cells were stained and the expression of indicated maturation markers was determined using flow cytometry analysis. Indicated is the average expression of CD80/86 ± SEM for cDC1 and cDC2 (*n* = 4-5). * p < 0.05, ** p <0.01, *** p < 0.005 and **** p < 0.0001, compared to control liposome.


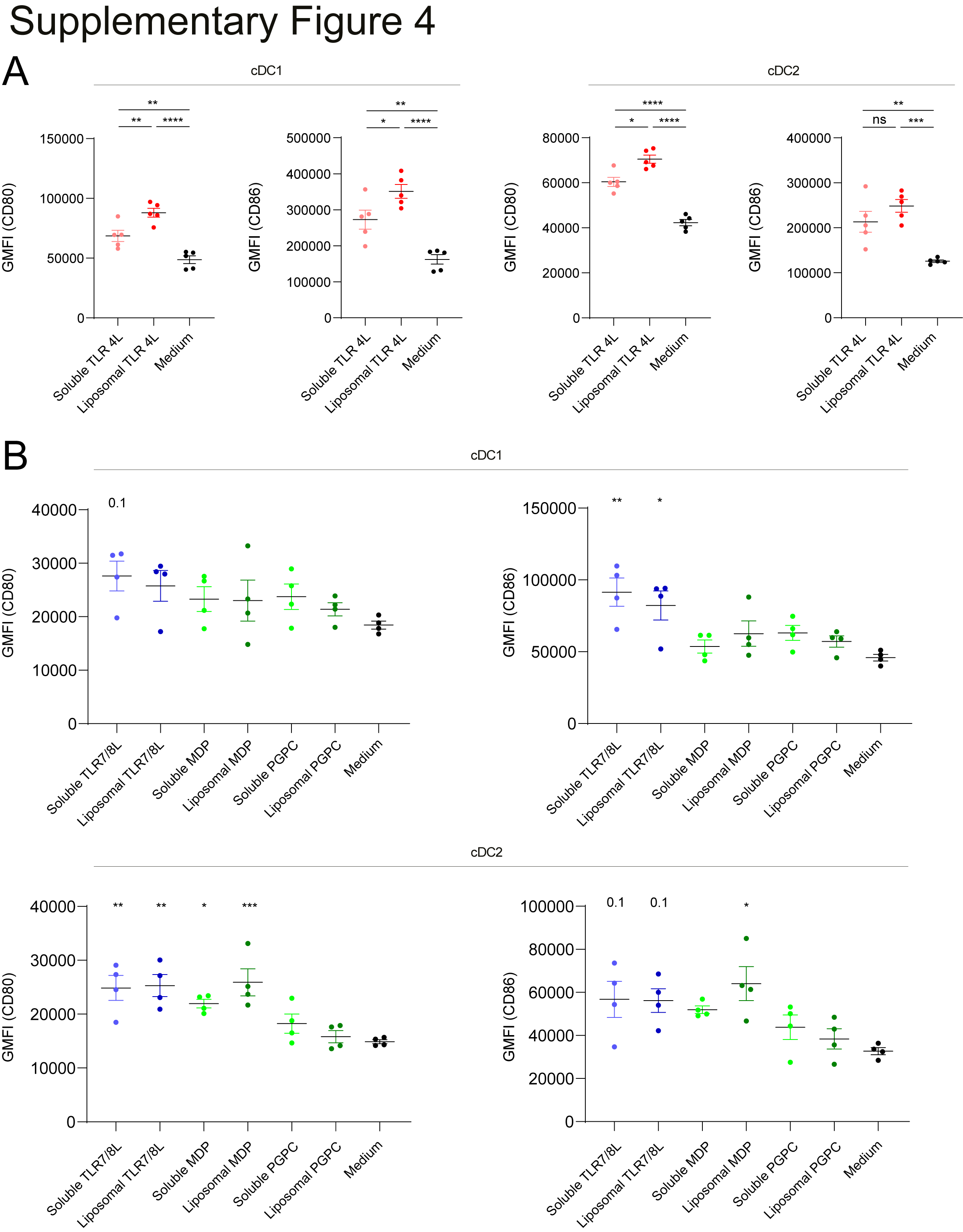


**Supplementary Figure 4: Liposomal and soluble TLR4L, TLR7/8L and MDP, but not PGPC, induce maturation of DC in vitro.** A/B) A single cell suspension of murine splenocytes was incubated with liposomes (100 uM phospholipid) or soluble adjuvant (dose equal to 2 mol% present in liposomes which is 5 µg/mL for TLR4L, 2 µg/mL for TLR7/8L, 2 µg/mL for PGPC or 3 µg/mL for MDP) for 45 minutes at 37 °C. Subsequently, cells were washed, cultured overnight and stained. The expression of indicated maturation markers was determined using flow cytometry analysis. Indicated is the average expression of CD80/86 ± SEM for cDC1 and cDC2 (*n* = 4-5), significance as indicated or compared to medium control. * p < 0.05, ** p <0.01, *** p < 0.005 and **** p < 0.0001.


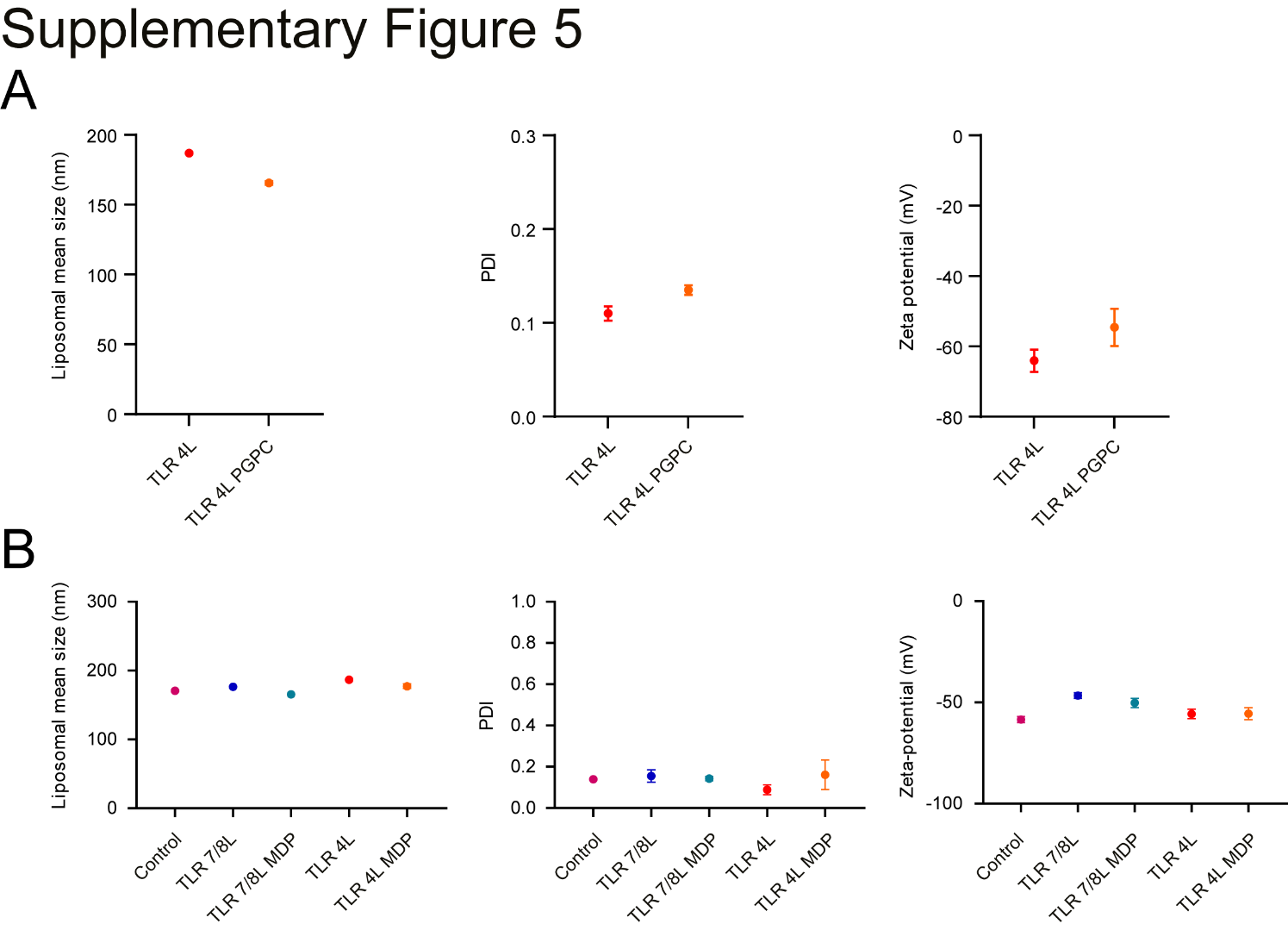


**Supplementary Figure 5:** **Characterization of liposomal batches related to Figure 3, 4, 5 and 6**. A/B) Antigen and TLRL and/or PGPC or MDP-containing liposomes were characterized by measurement of the liposomal mean size, PDI and zeta-potential, indicated is the average ± SEM of a technical triplicate.


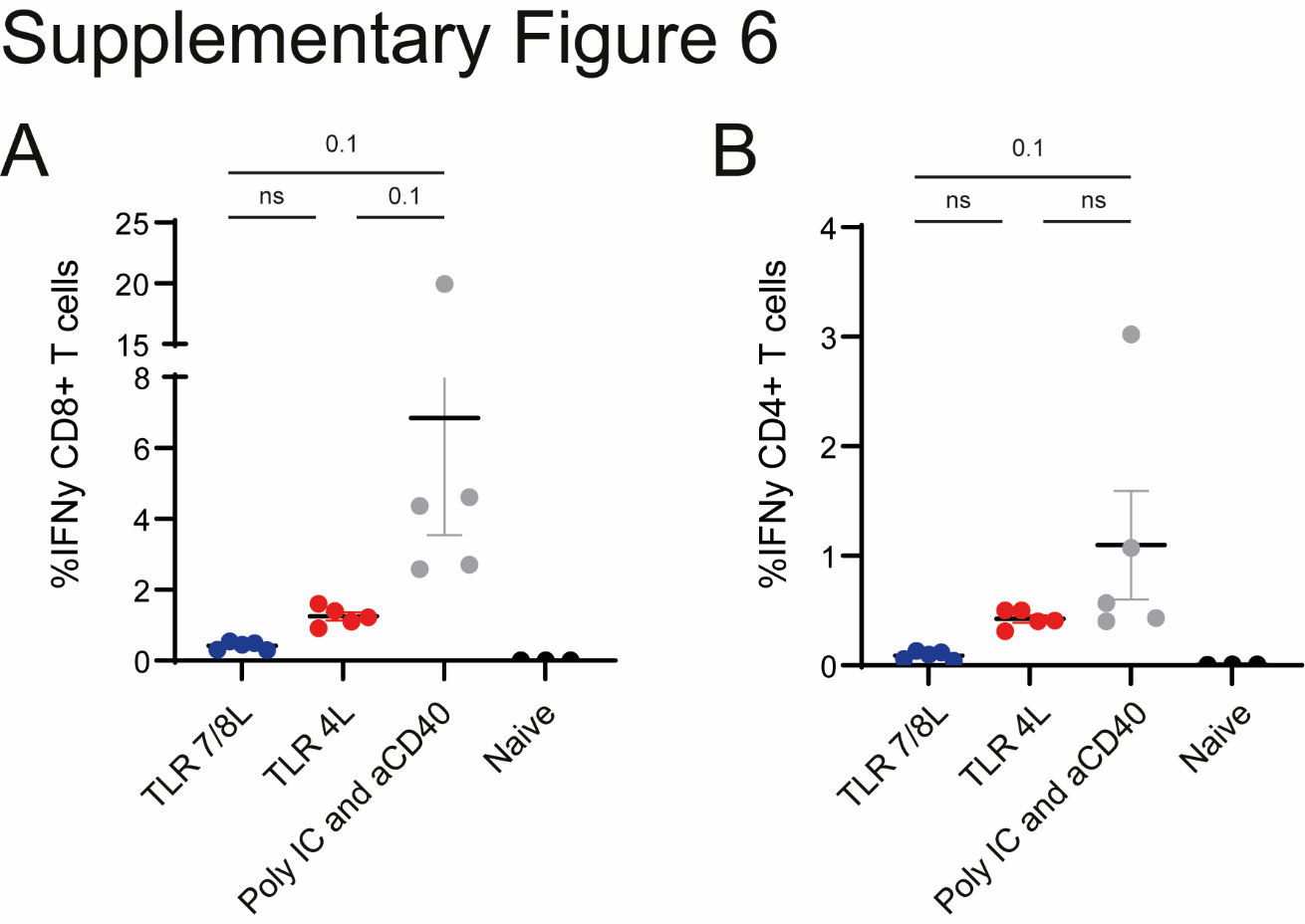


**Supplementary Figure 6: Immunization with antigen-containing GM3 liposomes supplemented with TLRL-containing GM3 liposomes elicit minor immune responses.** A/B) Mice were i.v. immunized with antigen-containing liposomes that were supplemented with various adjuvant-containing liposomes (1:1 ratio, both 22.5 nmol phospholipid), or antigen-containing liposomes that were supplemented soluble poly IC and aCD40 as positive control. After 7 days, spleens were collected and a single cell suspension was used for peptide restimulation for 5 or 25 hours for CD8^+^ and CD4^+^ T cells, respectively. Indicated is the average ± SEM of IFNγ-producing CD8^+^ and CD4^+^ T cells (A and B, respectively) (*n* = 3-5).

**
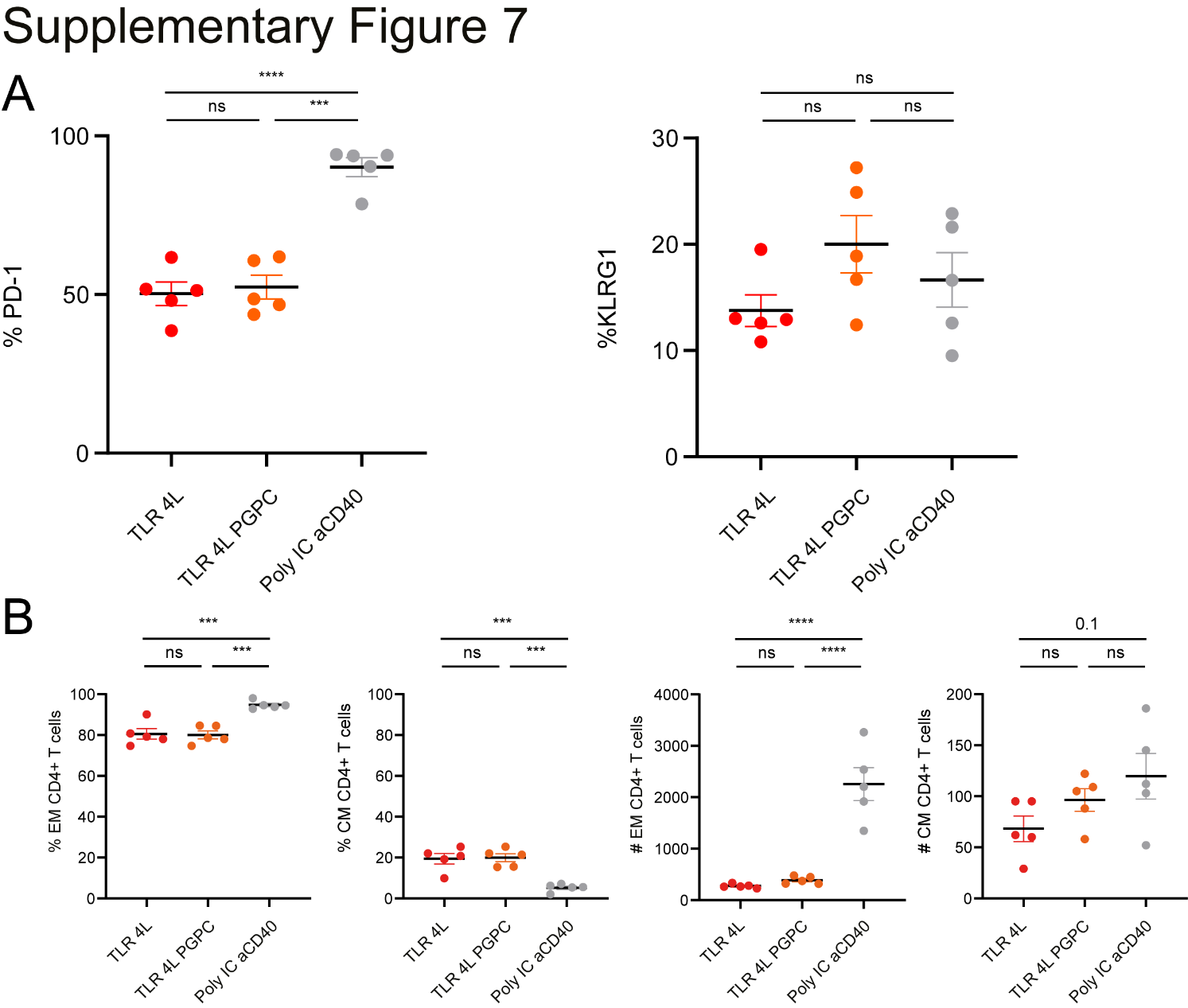
**

**Supplementary Figure 7: Phenotype of antigen-specific T cells after liposomal immunization.** A/B) Mice were i.v. injected with different liposome formulations, or antigen-containing liposomes with poly IC and aCD40 as soluble adjuvant. 7 days after injection, spleens were collected and a single cell suspension was used for tetramer staining. A) Indicated is the percentage of PD-1 or KLRG1 expressing H-2K^b^/OVA_257-264_ binding CD8^+^ T cells (*n* = 5). B) I-A^b^/OVA_262-276_ binding CD4^+^ T cells were subdivided in CD44^+^ Tem and CD44^+^CD62L^+^ Tcm cells. Indicated is the percentage (of antigen-specific CD4^+^ T cells) and number of cells identified as Tem or Tcm (based on 10^6^ events). Indicated is the average ± SEM (*n* = 5). *** p < 0.005 and **** p < 0.0001.


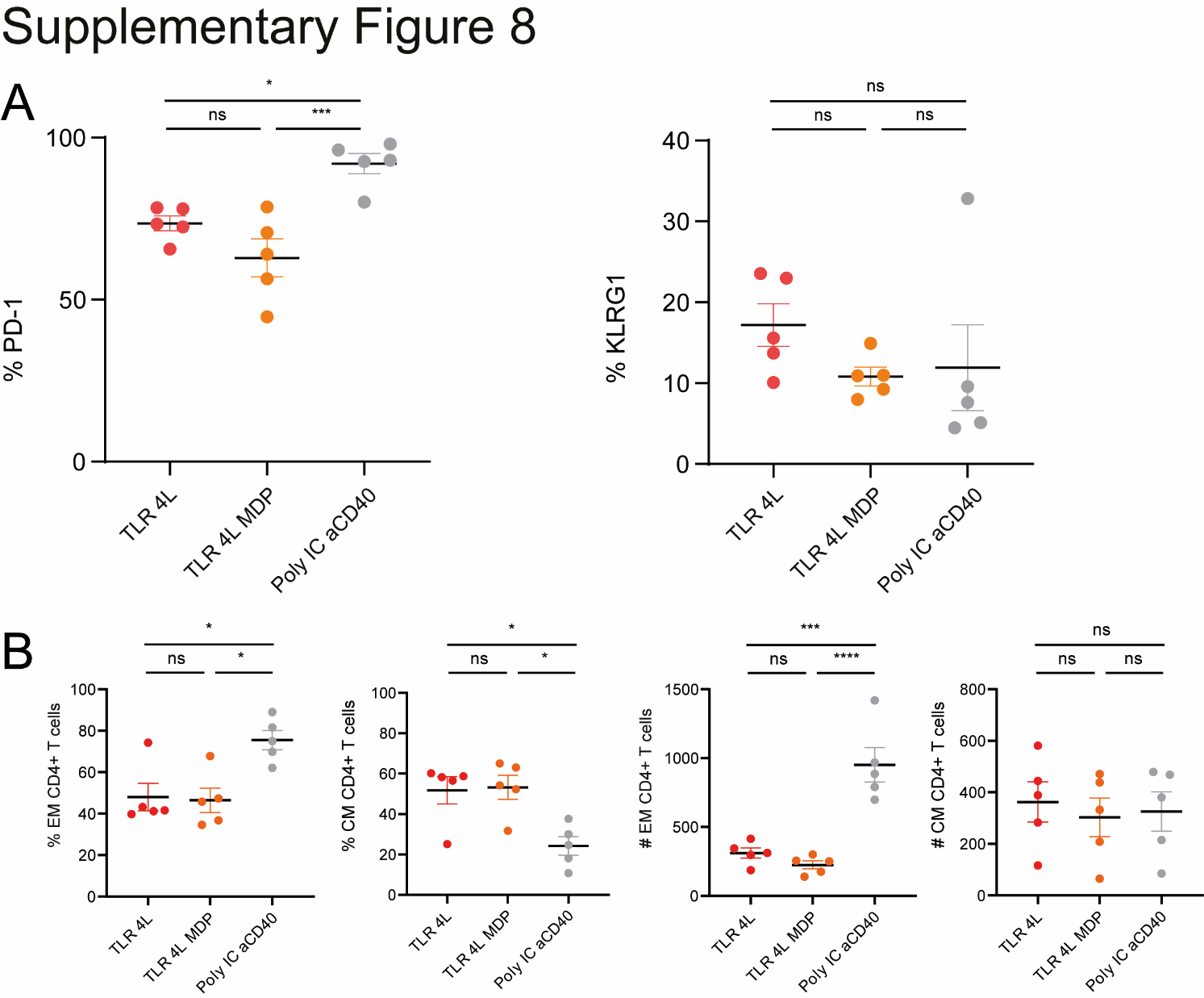


**Supplementary Figure 8: Phenotype of antigen-specific T cells after liposomal immunization.** A/B) Mice were injected with different liposome formulations, or antigen-containing liposomes with poly IC and aCD40 as soluble adjuvant. 7 days after injection, spleens were collected and a single cell suspension was used for tetramer staining. A) Indicated is the percentage of PD-1 or KLRG1 expressing H-2Kb/OVA_257-264_ binding CD8^+^ T cells (*n* = 5). B) I-Ab/OVA_262-276_ binding CD4^+^ T cells were subdivided in CD44^+^ Tem and CD44^+^CD62L^+^ Tcm cells. Indicated is the percentage (of antigen-specific CD4^+^ T cells) and number of cells identified as Tem or Tcm (based on 10^6^ events). Indicated is the average ± SEM (*n* = 5). * p < 0.05, *** p < 0.005 and **** p < 0.0001.
